# Synthesis of powdered and beaded chitosan materials modified with ZnO for removing lead (II) ions

**DOI:** 10.1038/s41598-022-22182-4

**Published:** 2022-10-13

**Authors:** Pimploy Ngamsurach, Nitthawan Namwongsa, Pornsawai Praipipat

**Affiliations:** 1 Department of Environmental Science, Khon Kaen University, Khon Kaen, 40002 Thailand; 2 Environmental Applications of Recycled and Natural Materials (EARN) Laboratory, Khon Kaen University, Khon Kaen, 40002 Thailand

**Keywords:** Engineering, Materials science

## Abstract

Lead contamination in wastewater may affect aquatic organisms, the environment, and human consumption because it is a highly toxic metal that caused human health effects. Thus, it is recommended to remove lead before releasing it into the environment. Powdered and beaded chitosan materials modified with ZnO were synthesized and investigated by various characterized techniques. Lead removal efficiencies of chitosan materials were studied by batch experiments, adsorption isotherms, and kinetics. Chitosan powder (CP), chitosan beads (CB), chitosan beads mixed ZnO (CZB), and chitosan beads coated ZnO (ZCB) were synthesized. CP represented a semi-crystalline structure while CB was an amorphous structure. CZB and ZCB were semi-crystalline structures with ZnO peaks. CP was a scaly-sheet and coarse surface while CB, CZB, and ZCB were sphere shapes with scaly-sheet surfaces. C, O, and N were the main chemical elements in chitosan materials, and Zn was detected in CZB and ZCB. O–H, N–H, and C–O were the main functional groups of chitosan materials. All chitosan materials had high lead removal efficiencies of more than 92%, and Freundlich and pseudo-second-order kinetic models well explained their adsorption patterns and mechanisms. Therefore, both adding metal oxide and changing material form are recommended for improving material efficiency, and ZCB was a good offer for further industrial applications.

## Introduction

The increasing of industries affects to release many pollutions into the environment by depending on the types of industries, so the pollutant sources can predict what kind of pollutant may release and affect the environment. Several industries such as the battery, petroleum, and pigment industries use lead as a raw material^1^, so lead may discharge into wastewater. Lead is one of the highly toxic metals which its toxicity affects to malfunction of main human systems^2^, and its accumulation in humans might be from food chains or using lead-contaminated water. Therefore, the contaminated lead in wastewater is highly recommended to remove below 0.2 mg/L following USEPA standards for a safe environment including human health.

Several methods are used for lead removal from wastewater such as chemical precipitation, ion exchange, oxidation–reduction, reverse osmosis, ultra-filtration, and adsorption^3,4^; however, those methods have both advantages and disadvantages which need to weigh before use. The operation and disposal costs are principal factors for a decision, and a reasonable cost with an effective method is a priority preferred. Then, an adsorption method is an interesting method to apply for lead removal from wastewater^5,6^. A key factor of the adsorption method is the selection of adsorbent, and the removal efficiency depends upon the specific adsorbent which matches the target pollutants. In addition, one of considering factors is a low-cost adsorbent, so the perfect adsorbent should be effective with a proper cost. In previous studies, various adsorbents from nature, commerce, and waste have been used for removing heavy metals such as zeolite, kaolin, resin, sawdust, fruit wastes, and food wastes^7,8^. Since the increase in human population results in an increase in food consumption, it affects the increase of food waste as well. Therefore, the recycling of food waste is an interesting idea to obtain two benefits of waste management and the use of recycled wastes. Using animals with shells and carapaces such as mollusk shells, shrimp shells, and carapaces of crab^9,10^ as raw materials for chitosan synthesis is an alternative option for reducing food wastes and using the benefit of chitosan as heavy metal adsorbents. Many studies have reported chitosan could remove various heavy metals from wastewater such as cadmium (Cd), copper (Cu), chromium (Cr), nickel (Ni), arsenic (As), mercury (Hg), and lead (Pb)^9–14^; however, the idea of improving chitosan efficiency for specific removing of a target pollutant in natural water or wastewater is interesting to investigate how to improve it.

Many articles have used various metal oxides such as iron oxide (FeOH), titanium dioxide (TiO_2_), aluminum oxide (Al_2_O_3_), manganese oxide (MnO), and zinc oxide (ZnO) to improve heavy metal adsorbents because they promote the increase of surface area and pore volume of adsorbents resulting in the increase of active sites for adsorbing heavy metal ions. As a result, the addition of those metal oxides into adsorbents helps to increase the adsorbent efficiency for removing metal ions^15,16^. However, metal oxides are not directly used because of problems of clogging, pressure drop, and difficult separation after treatments in real industrial applications of continuous flow system^17^, so adding metal oxide into an efficient adsorbent may be a good idea to promote a high adsorbent efficiency for removing specific target pollutant. In addition, the development of a durable adsorbent form may help for long-term use and save operation costs as well. Therefore, this study attempts to synthesize chitosan materials by adding metal oxide and changing material form to improve chitosan material efficiencies for lead removal from wastewater for further industrial applications.

This study aimed to synthesize four chitosan materials which were chitosan powder (CP), chitosan beads (CB), chitosan beads mixed ZnO (CZB), and chitosan beads coated ZnO (ZCB) for lead removal from the synthetic wastewater. Their characterizations of the size of surface area, pore volume, pore size, crystalline formation, surface area, chemical elements, and functional groups were investigated through Brunauer–Emmett–Teller (BET), X-ray Diffractometer (XRD), Field Emission Scanning Electron Microscopy and Focus Ion Beam (FESEM-FIB), Energy Dispersive X-ray Spectrometer (EDX), and Fourier Transform Infrared Spectroscopy (FTIR), respectively. Moreover, a series of batch experiments were conducted to compare their lead removal efficiencies, and adsorption isotherms and kinetics were also examined to understand their adsorption patterns and mechanisms.

## Results and discussion

### The physical characteristics of chitosan materials

**Figure 1:**
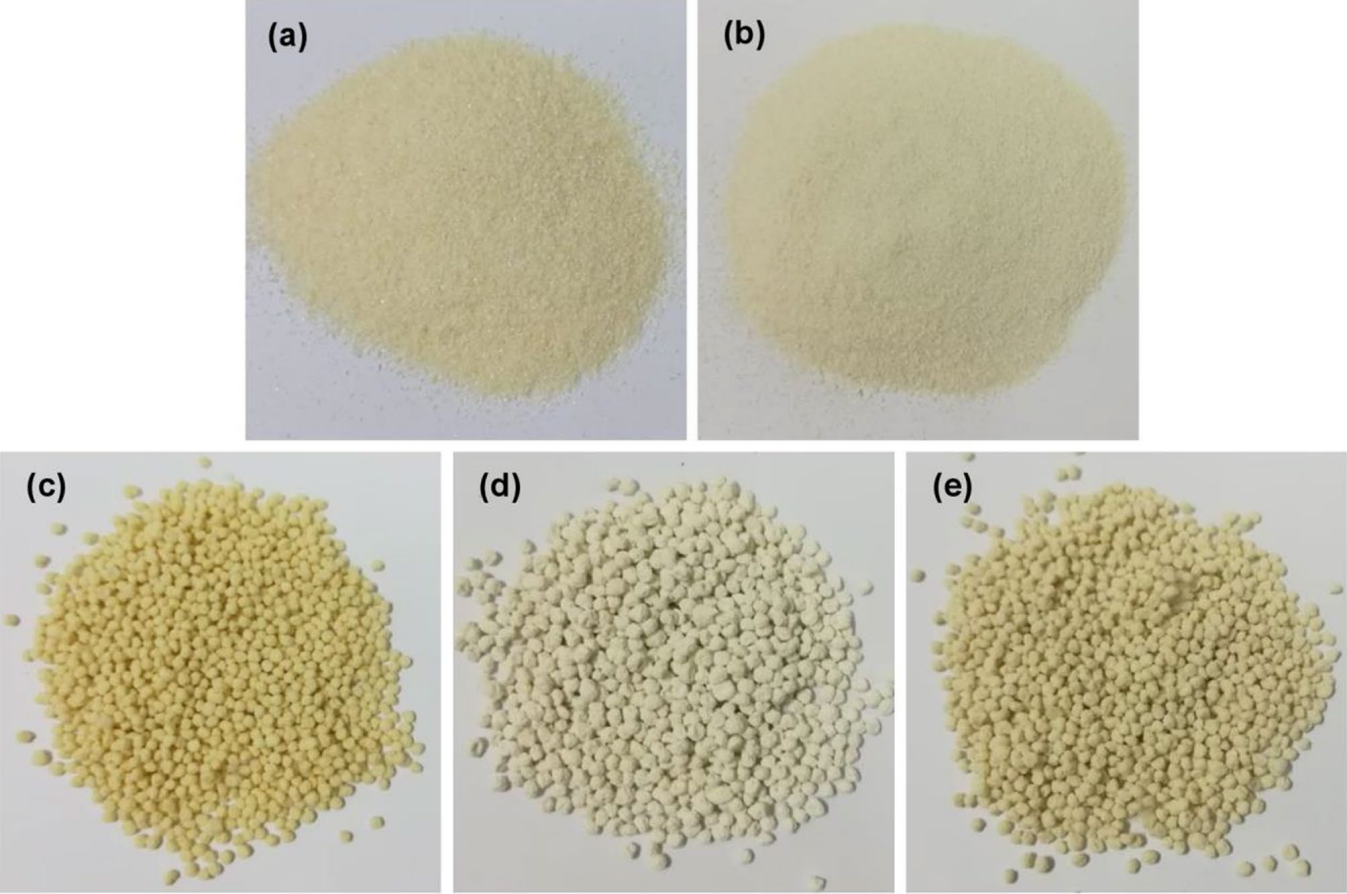
Physical characteristics of (**a**) chitosan standard, (**b**) chitosan powder (CP), (**c**) chitosan beads (CB), (**d**) chitosan beads mixed ZnO (CZB), and (**e**) chitosan beads coated ZnO (ZCB).

The physical characteristics of all chitosan materials are demonstrated in Fig. 1a-e. CP was fine yellow powdered color similar to the chitosan standard shown in Fig. 1a,b. For chitosan bead materials, CB was a yellow beaded color which had the same color as the chitosan powder reported in Fig. 1c. For CZB and ZCB, they were a lightly yellow beaded color whereas ZCB demonstrated a darker color than CZB illustrated in Fig. 1d,e.

### Characterizations of chitosan materials

#### Physicochemical properties

The physicochemical properties of all chitosan materials by BET analysis are reported in Table 1. The sizes of the surface area of chitosan standard, CP, CB, CZB, and ZCB were 12.30, 11.85, 12.46, 13.11, and 13.54 m^2^/g, respectively which ZCB presented a higher surface area than other chitosan materials. For pore volumes, they were 0.010, 0.011, 0.013, 0.016, and 0.018 cm^3^/g for chitosan standard, CP, CB, CZB, and ZCB, respectively which ZCB demonstrated a higher pore volume than others. The pore sizes of chitosan standard, CP, CB, CZB, and ZCB were 3.905, 4.024, 3.897, 3.745, and 3.626 nm, respectively which ZCB represented a smaller pore size than other chitosan materials. Moreover, pore sizes of all chitosan materials were classified to a mesoporous size (2–50 nm) followed by the classification of IUPAC^18^. As a result, the synthesized chitosan powder (CP) had the size of surface area, pore volume, and pore size close to the chitosan standard and similarly reported by previous studies of chitosan synthesis from shrimp shells^19,20^. Moreover, the addition of ZnO increased the surface area and pore volume while the pore size decreased. In principle, the high heavy metal removal will be found in the adsorbent with a high surface area and small pore size^21^. Therefore, since ZCB had a higher surface area and smaller pore size than others, ZCB might better adsorb or remove lead than other chitosan materials.

**Table 1:** The sizes of surface area, pore volumes, and pore sizes of chitosan standard, chitosan powder (CP), chitosan beads (CB), chitosan beads mixed ZnO (CZB), and chitosan beads coated ZnO (ZCB) by BET analysis.

Material	Surface area (m^2^/g)*	Pore volume (cm^3^/g)**	Pore size (nm)***
Chitosan standard	12.30	0.010	3.905
CP	11.85	0.011	4.024
CB	12.46	0.013	3.897
CZB	13.11	0.016	3.745
ZCB	13.54	0.018	3.626

*BJH method cumulative adsorption surface area.

**BJH method cumulative adsorption pore volume.

***BJH method adsorption pore radius.

#### XRD

**Figure 2:**
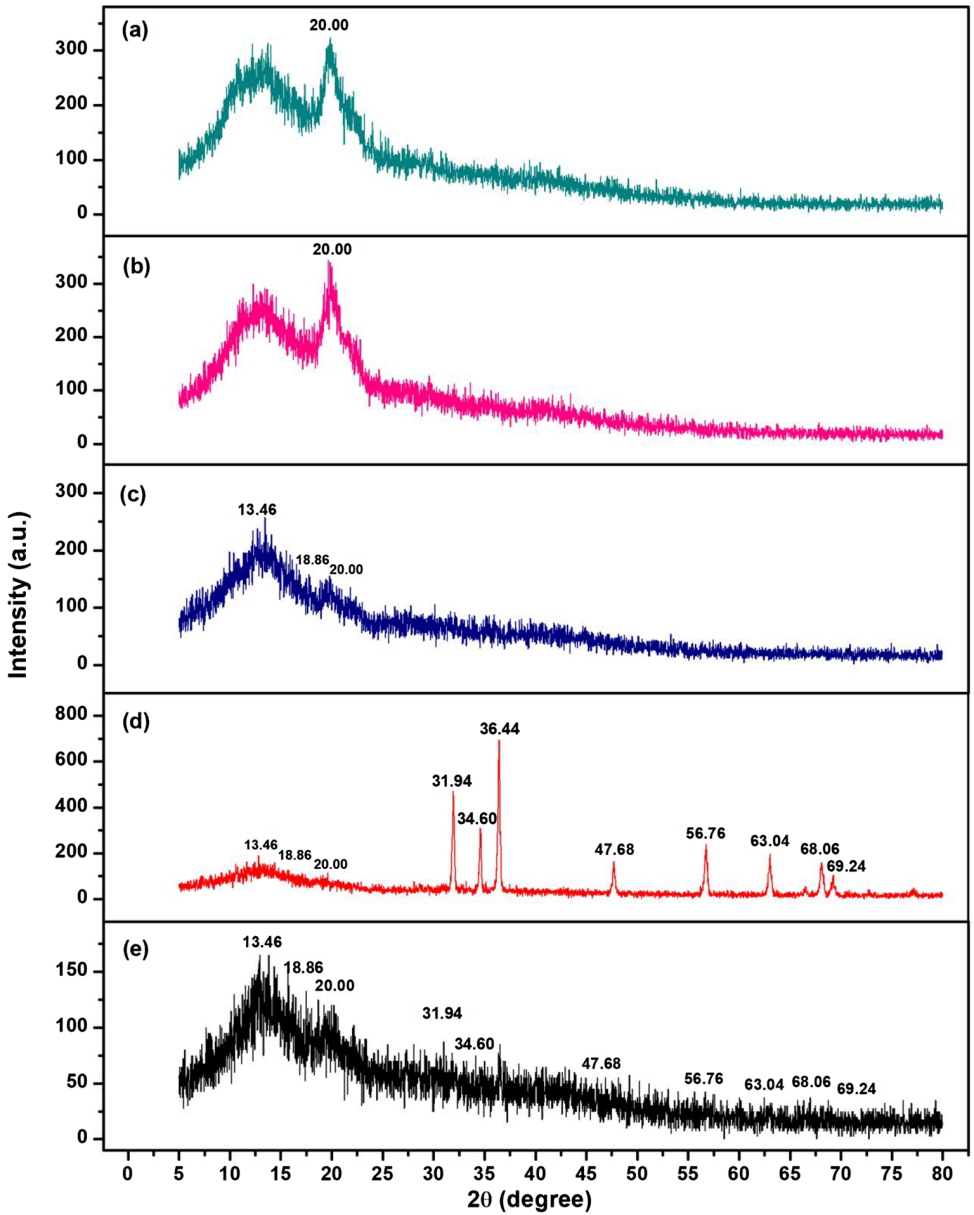
Crystalline structures of (**a**) chitosan standard, (**b**) chitosan powder (CP), (**c**) chitosan beads (CB), (**d**) chitosan beads mixed ZnO (CZB), and (**e**) chitosan beads coated ZnO (ZCB).

X-ray Diffraction (XRD) technique was used to identify the crystalline formations of all chitosan materials, and the results are demonstrated in Fig. 2a-e. CP represented the semi-crystalline structure which demonstrated the specific chitosan peak at approximately 2 $\uptheta$ of 20.00º matched to the chitosan standard^22,23^ shown in Fig. 2a,b. Figure 2c represented the amorphous structure of CB with specific alginate peaks of 13.46° and 18.86°^24,25^, and Fig. 2d,e illustrated the semi-crystalline structures with the specific sodium alginate similar to CB and ZnO peaks of CZB and ZCB. CZB represented the specific ZnO peaks of 31.94°, 34.60°, 36.44°, 47.68°, 56.76°, 63.04°, 68.06°, and 69.24° which related to hexagonal phases of ZnO following JCPDS:36–1451^26^ whereas ZCB demonstrated the low intensity of specific ZnO peaks with the amorphous structure than CZB. As a result, it could be confirmed the addition of ZnO into CZB and ZCB, and the method of adding ZnO into CZB and ZCB might affect the intensity of ZnO peaks. For CZB, ZnO was added into the chitosan powder (CP) before forming beads which this method might support the homogeneous mix of chitosan powder and ZnO resulting in the high intensity of ZnO peaks. While ZCB was synthesized from coating ZnO on the chitosan beads (CB), this method might result in the low intensity of ZnO peaks. Since ZCB was synthesized from CB before adding ZnO, its XRD pattern was mainly dominated by the XRD pattern of CB which presented the amorphous structure.

#### FESEM-FIB

**Figure 3:**
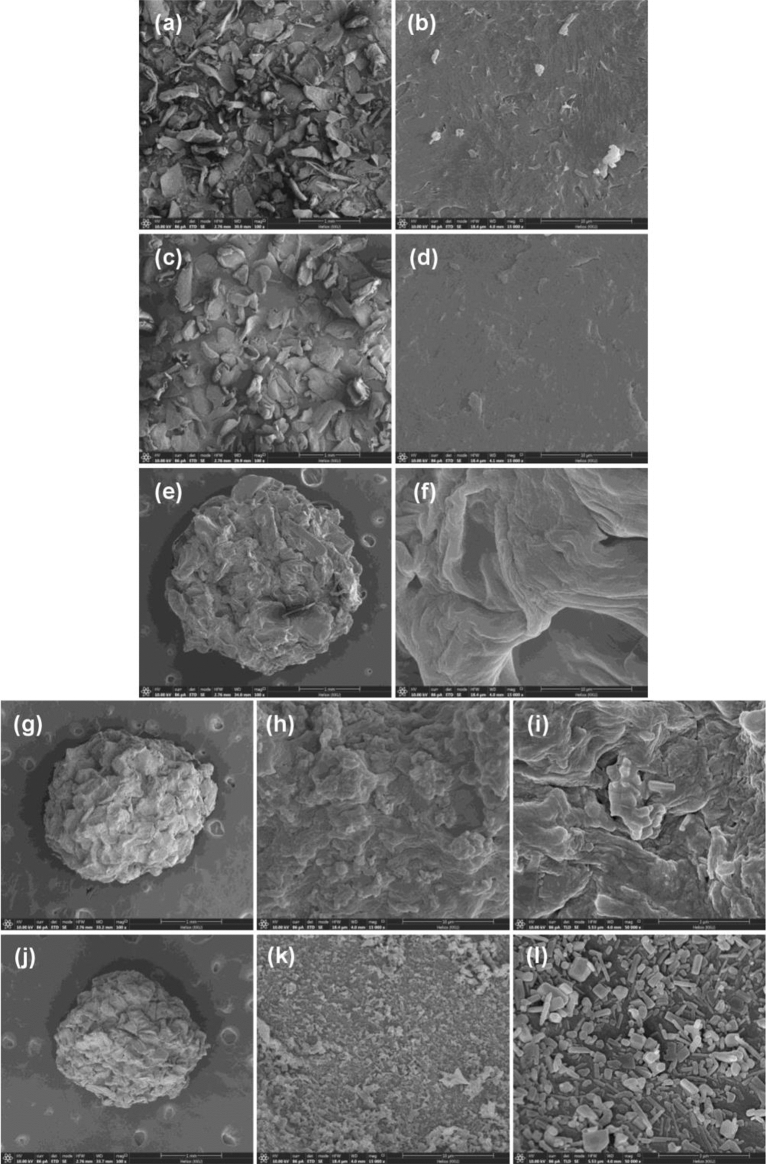
FESEM-FIB images of surface morphology of (**a**, **b**) chitosan standard, (**c**, **d**) chitosan powder (CP), (**e**, **f**) chitosan beads (CB), (**g**, **h**, **i**) chitosan beads mixed ZnO (CZB), and (**j**, **k**, **l**) chitosan beads coated ZnO (ZCB).

The surface morphologies of all chitosan materials were investigated by FESEM-FIB analysis, and their results are demonstrated in Fig. 3a-l. CP was a scaly-sheet surface at 100X magnification with 1 mm shown in Fig. 3c whereas it represented a coarse surface at 15,000X magnification with 10 µm reported in Fig. 3d which these results were similar to the chitosan standard illustrated in Fig. 3a,b. CB had a sphere shape with a scaly-sheet surface at 100X magnification with 1 mm shown in Fig. 3e, and it represented a layer-rough surface at 15,000X magnification with 10 µm shown in Fig. 3f. For CZB and ZCB, they also had sphere shapes with scaly-sheet surfaces at 100X magnification with 1 mm shown in Fig. 3g,j. CZB was an unsmooth surface grouped whereas ZCB was a flake-rough surface at 15,000X magnification with 10 µm shown in Fig. 3h,k. When they were zoomed at 50,000X magnification with 3 µm, it might be evidence of ZnO grouping with chitosan in CZB whereas ZCB demonstrated the specific ZnO rod spread around the ZCB surface shown in Fig. 3i,l. As a result, this study was successfully adding of ZnO into CZB and ZCB which corresponded to XRD results.

#### EDX

The chemical compositions of all chitosan materials were analyzed by EDX and reported in Table 2, and three main chemical elements of carbon (C), oxygen (O), and nitrogen (N) were detected. Other elements of sodium (Na), calcium (Ca), and chloride (Cl) were observed in all chitosan beads whereas zinc (Zn) was only found in chitosan beads with adding ZnO. In Table 2, the chemical compositions of CP were closely valued to the chitosan standard, so it could be confirmed the ability to synthesize chitosan in this study. For CB, Na, Ca, and Cl were detected which might be from using sodium alginate and calcium chloride in a bead formation process. In addition, C and O were decreased when the material form was changed. For CZB and ZCB, their C and O were decreased while Na, Ca, and Cl were also detected similarly to the CB result. Moreover, the percentage by weight of Zn in ZCB had higher than CZB corresponding to the FESEM-FIB result which found ZnO rod on the surface. As a result, this study was successful to add ZnO into CZB and ZCB.

**Table 2:** The chemical compositions of chitosan standard, chitosan powder (CP), chitosan beads (CB), chitosan beads mixed ZnO (CZB), and chitosan beads coated ZnO (ZCB) by EDX analysis.

Material	Chemical element (%wt)						
	C	O	N	Na	Ca	Cl	Zn
Chitosan standard	53.5	41.7	4.8	–	–	–	–
CP	52.0	42.8	5.2	–	–	–	–
CB	45.5	41.4	4.9	0.8	5.8	1.6	–
CZB	30.6	20.3	5.0	0.9	5.4	1.3	36.5
ZCB	30.3	11.8	5.1	1.0	5.2	1.4	45.2

#### FTIR

**Figure 4:**
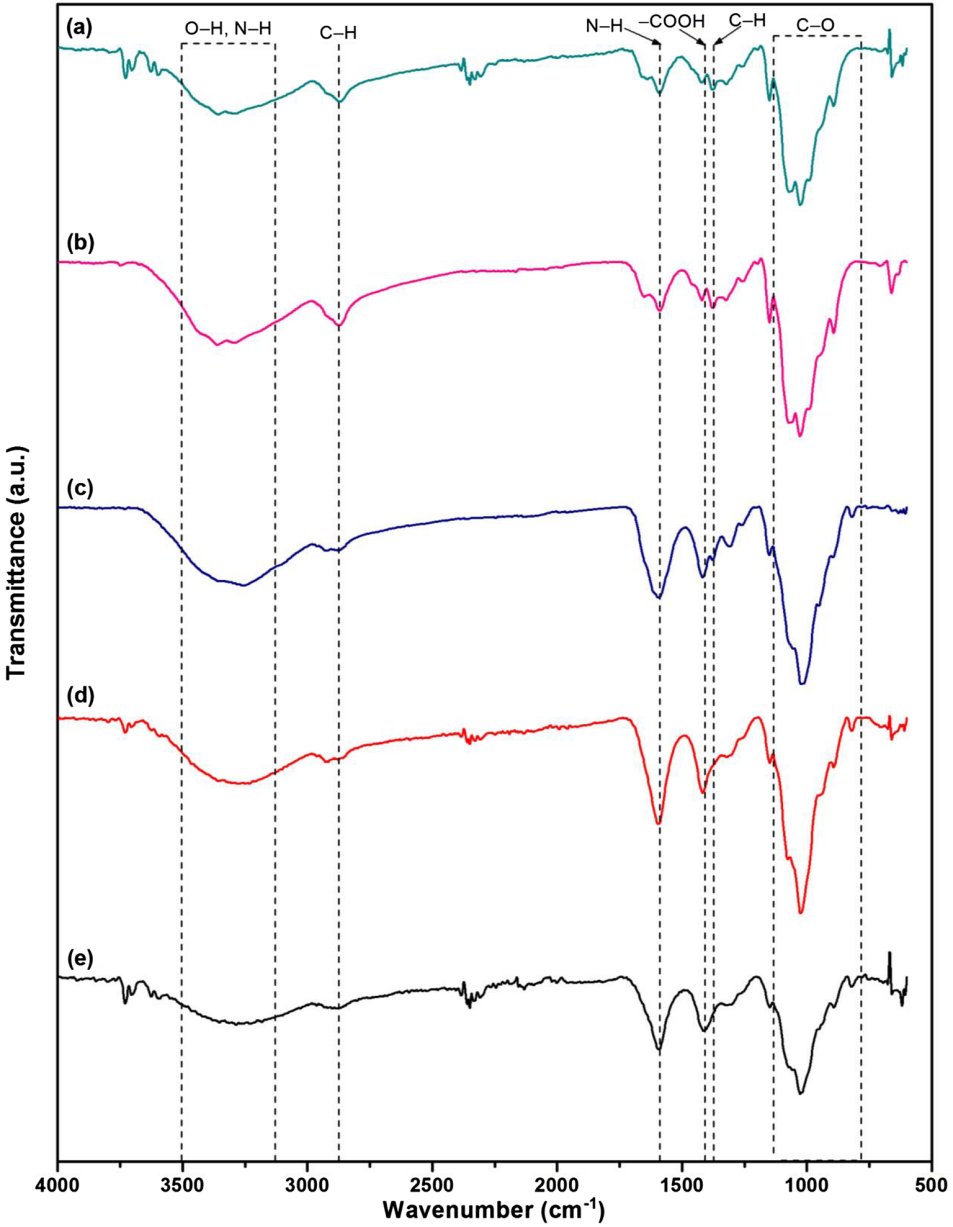
FTIR spectra of (**a**) chitosan standard, (**b**) chitosan powder (CP), (**c**) chitosan beads (CB), (**d**) chitosan beads mixed ZnO (CZB), and (**e**) chitosan beads coated ZnO (ZCB).

The chemical functional groups of all chitosan materials were classified by FTIR analysis, and their results are illustrated in Fig. 4a-e. Three main chemical function groups of O–H, N–H, and C–O were detected in all chitosan materials. O–H and N–H presented polysaccharide molecules, amide or amine groups of chitosan, and C–O represented the carbohydrate structure of chitosan. In addition, C–H was CH_3_ and CH_2_ in a chitosan polymer^23,27–29^. For the chitosan standard, the stretching of O–H and N–H at 3354.65 cm^-1^, stretching of C–H at 2871.78 cm^-1^, stretching of N–H at 1589.95 cm^-1^, blending of C–H at 1376.17 cm^-1^, C–O at 1026.94 cm^-1^, and glucose ring stretching at 893.14 cm^-1^ were observed shown in Fig. 4a. For CP, it detected the stretching of O–H and N–H at 3359.41 cm^-1^, stretching of C–H at 2873.50 cm^-1^, stretching of N–H at 1589.29 cm^-1^, blending of C–H at 1377.20 cm^-1^, C–O at 1027.72 cm^-1^, and glucose ring stretching at 892.71 cm^-1^ which was similarly corresponded to chemical functional groups of chitosan standard shown in Fig. 4b. For CB, the stretching O–H and N–H at 3255.99 cm^-1^, stretching of N–H at 1592.82 cm^-1^, N–H interacted to –COOH at 1416.98 cm^-1^ represented the sodium alginate in the chitosan material^30,31^, blending of C–O at 1022.59 cm^-1^, and glucose ring stretching at 819.62 cm^-1^ were found shown in Fig. 4c. For CZB, it demonstrated the stretching of O–H and N–H at 3274.74 cm^−1^, stretching of N–H of 1593.94 cm^−1^, N–H interacted to –COOH at 1416.85 cm^−1^ represented the sodium alginate in the chitosan material, and C–O at 1024.79 cm^−1^ shown in Fig. 4d. Finally, ZCB was displayed the stretching of O–H and N–H at 3284.74 cm^−1^, stretching of N–H at 1592.34 cm^−1^, N–H interacted to –COOH at 1413.20 cm^−1^ represented the sodium alginate in the chitosan material, and C–O at 1026.75 cm^−1^ shown in Fig. 4e.

In comparison, the chitosan powder (CP) found four chemical functional groups of O–H, N–H, C–H, and C–O whereas chitosan beads of CB, CZB, and ZCB detected four chemical functional groups of O–H, N–H, C–O, and –COOH. As a result, the changing material form from powder to bead form resulted in the disappearance of C–H, and the carboxyl groups (–COOH) of sodium alginate were found in all chitosan beads. The connection between sodium alginate and chitosan might be from the electrostatic interaction of carboxyl groups (–COOH) of sodium alginate with the amino or ammonium groups (–NH_2_) of chitosan^19,32^. In addition, the addition of ZnO in CZB and ZCB might be the connection of ZnO with N–H or O–H in the chitosan similarly reported by M.M. AbdElhady^33^.

### Batch experiments

#### The effect of dose

**Figure 5:**
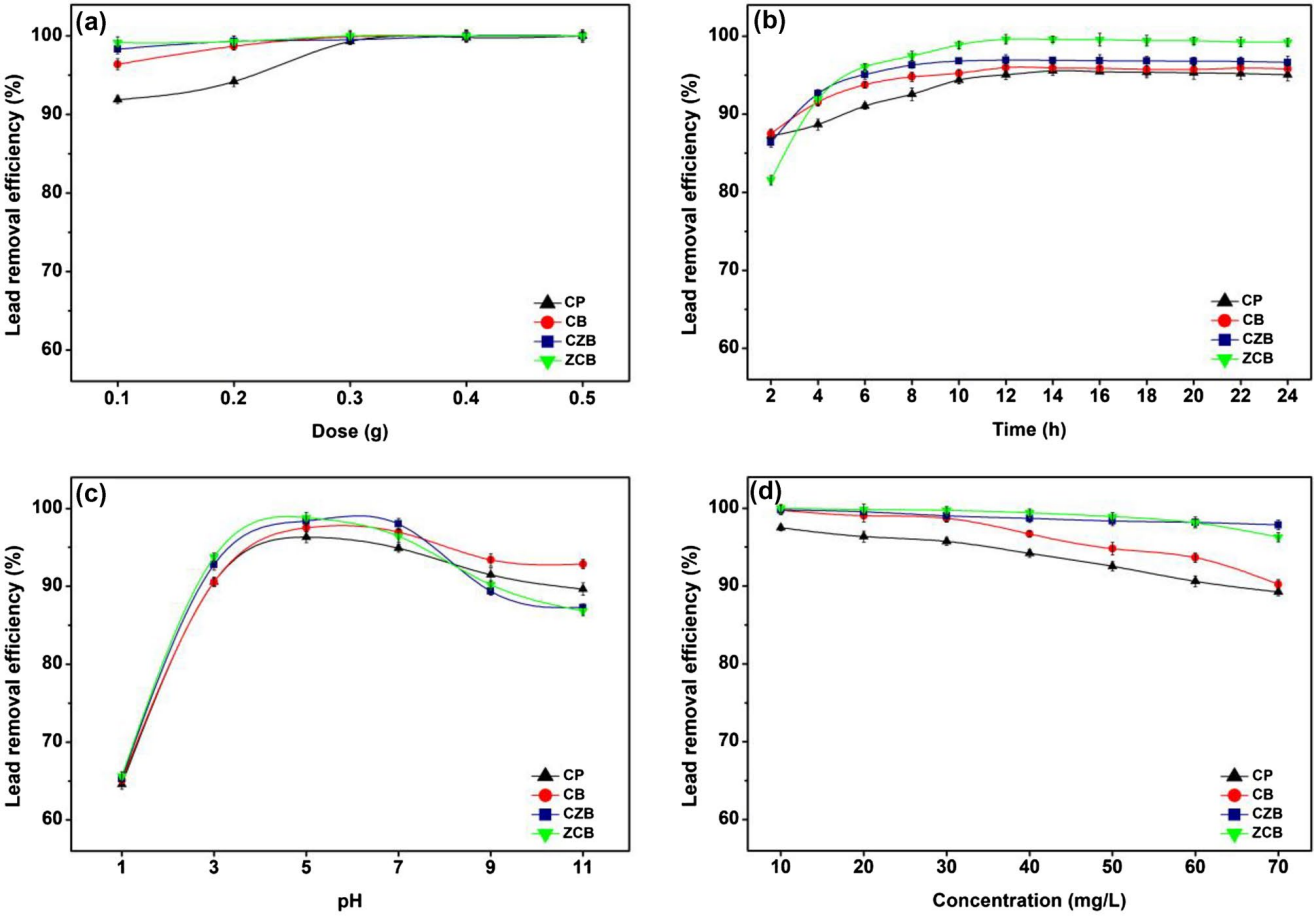
The batch experiments of chitosan powder (CP), chitosan beads (CB), chitosan beads mixed ZnO (CZB), and chitosan beads coated ZnO (ZCB) in (**a**) dose, (**b**) contact time, (**c**) pH, (**d**) concentration for lead adsorptions.

The effect of dose from 0.1 to 0.5 g of CP, CB, CZB, and ZCB on lead removal efficiencies with the control condition of the initial lead concentration of 50 mg/L, a sample volume of 200 mL, a contact time of 24 h, pH 5, and a shaking speed of 150 rpm shown in Fig. 5a. Lead removal efficiencies of all chitosan materials were increased with increasing of material dosage because of the increasing of adsorption sites in chitosan materials^11^. The highest lead removal efficiencies of 100% were found at 0.5, 0.4, 0.4, and 0.3 g for CP, CB, CZB, and ZCB, respectively. Therefore, they were used as the optimum dosage of all chitosan materials for studying the contact time effect.

#### The effect of contact time

The effect of contact time from 2 to 24 h of CP, CB, CZB, and ZCB on lead removal efficiencies with the control condition of the initial lead concentration of 50 mg/L, a sample volume of 200 mL, pH 5, a shaking speed of 150 rpm, and the optimum dosage demonstrated in Fig. 5b. Lead removal efficiencies on chitosan materials were increased with increasing of contact time from 2 to 24 h, and the highest lead removal efficiency with a constant contact time represented the equilibrium lead adsorption^13^. The contact time of 14, 12, 12, and 12 h demonstrated the highest lead removal efficiencies of 95.55, 95.97, 96.92, and 99.60% for CP, CB, CZB, and ZCB, respectively. Therefore, those dosages and contact time were used as the optimum dosage and contact time of all chitosan materials for investigating the pH effect.

#### The effect of pH

The pH effect of 1, 3, 5, 7, 9, and 11 of CP, CB, CZB, and ZCB on lead removal efficiencies with the control condition of the initial lead concentration of 50 mg/L, a sample volume of 200 mL, a shaking speed of 150 rpm, and the optimum dosage and contact time displayed in Fig. 5c. Lead removal efficiencies of all chitosan materials were increased with increasing of pH from 1 to 5, and then they were decreased from pH 7 to 11. Lead precipitations in alkaline pH might affect the decrease of lead adsorptions of chitosan materials^34^. In addition, the previous studies reported the pH_pzc_ of various chitosan materials for lead adsorptions were found at a pH solution higher than pH 4^35–37^. In this study, the highest lead removal efficiencies of 96.30, 97.51, 98.40, and 98.81% for CP, CB, CZB, and ZCB, respectively were found at pH 5, so pH 5 was the optimum pH of all chitosan materials. Therefore, those dosages, contact time, and pH were used as the optimum dosage, contact time, and pH of all chitosan materials for examining the concentration effect.

#### The effect of concentration

The effect of concentration from 10 to 70 mg/L of CP, CB, CZB, and ZCB on lead removal efficiencies with the control condition of a sample volume of 200 mL, a shaking speed of 150 rpm, and the optimum dosage, contact time, and pH illustrated in Fig. 5d. Lead removal efficiencies from 10 to 70 mg/L were 89.24–97.49%, 93.00–98.77%, 98.46–98.85%, and 97.34–99.85% for CP, CB, CZB, and ZCB, respectively which they were decreased with increasing lead concentration. In principle, the active sites of adsorbent are decreased with increasing initial lead concentration resulting in a decrease in lead removal efficiency^12^. For the initial lead concentration of 50 mg/L, their lead removal efficiencies were 92.53, 94.81, 98.35, and 98.91% for CP, CB, CZB, and ZCB, respectively, and ZCB demonstrated the highest lead removal efficiency than others. Therefore, all chitosan materials were high-quality adsorbents for lead removal at 50 mg/L from wastewater more than 92%.

Finally, 0.5 g, 14 h, pH 5, 50 mg/L, 0.4 g, 12 h, pH 5, 50 mg/L, 0.4 g, 12 h, pH 5, 50 mg/L, and 0.3 g, 12 h, pH 5, 50 mg/L were the optimum conditions in dose, contact time, pH, and concentration of CP, CB, CZB, and ZCB, respectively, and they could be arranged in order from high to low of ZCB > CZB > CB > CP. Therefore, chitosan materials could be increased their lead removal efficiencies by adding metal oxide and changing material form similarly found in another study^21^, and ZCB demonstrated the highest lead removal efficiency than other chitosan materials.

### Isotherm study

**Figure 6:**
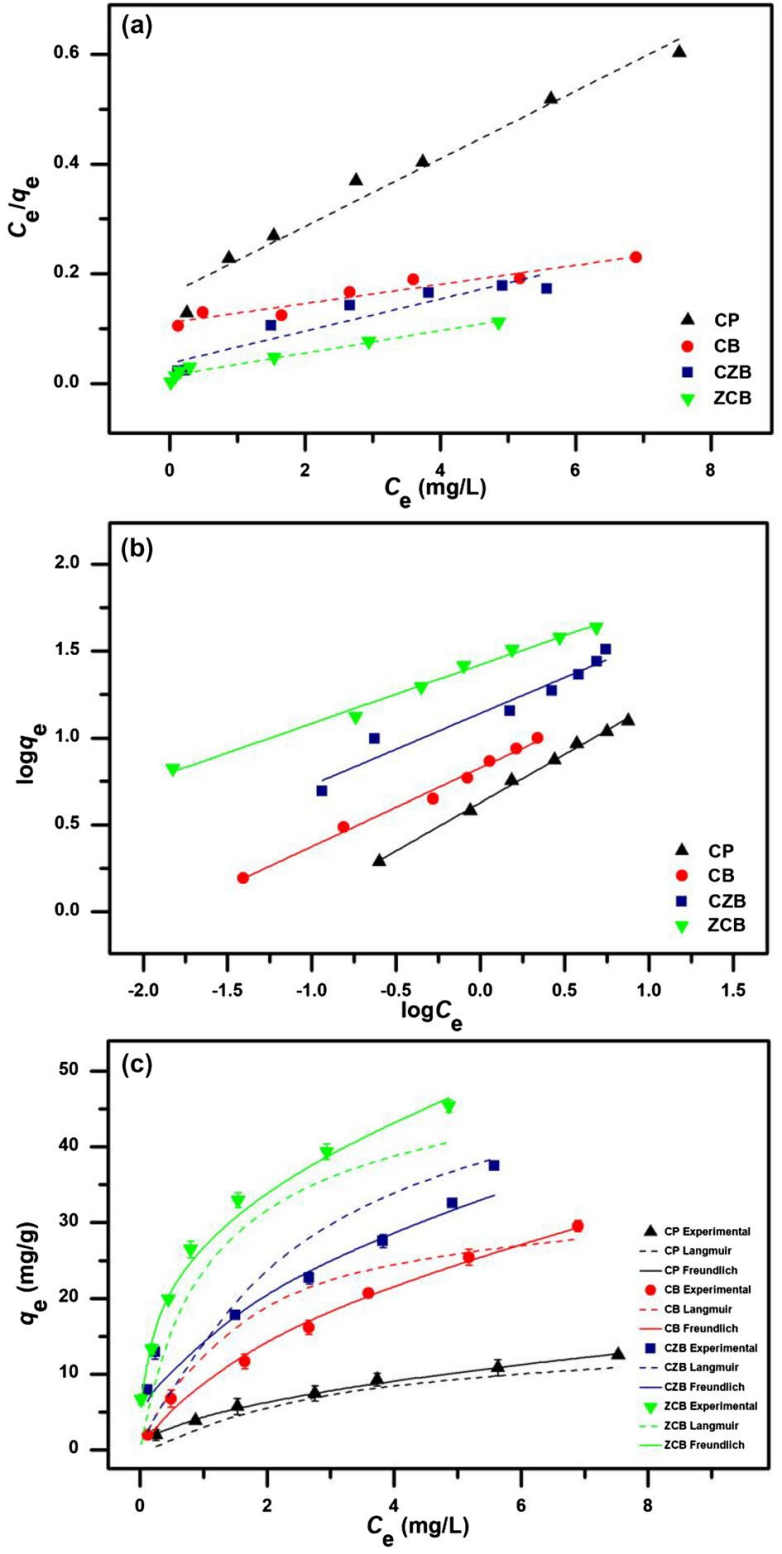
(**a**) Linear Langmuir, (**b**) Linear Freundlich, and (**c**) nonlinear adsorption isotherms of chitosan powder (CP), chitosan beads (CB), chitosan beads mixed ZnO (CZB), and chitosan beads coated ZnO (ZCB) for lead adsorptions.

The isotherm study was designed to know the adsorption patterns of all chitosan materials by using linear and nonlinear Langmuir and Freundlich models. Linear Langmuir and Freundlich isotherms were plotted by *C*_e_*/q*_e_ versus *C*_e_ and log *q*_e_ versus log *C*_e_, respectively whereas nonlinear Langmuir and Freundlich isotherms were plotted by *C*_e_ versus *q*_e_. The results are demonstrated in Fig. 6a-c, and the equilibrium isotherm parameters are reported in Table 3.

**Table 3:** The equilibrium isotherm parameters of chitosan powder (CP), chitosan beads (CB), chitosan beads mixed ZnO (CZB), and chitosan beads coated ZnO (ZCB) for lead adsorptions.

Model	Regression method	Parameters	CP	CB	CZB	ZCB
Langmuir isotherm	Linear	*q*_m_ (mg/g)	16.207	39.216	41.041	47.349
		*K*_L_ (L/mg)	0.378	0.770	0.383	0.760
		*R*^2^	0.971	0.962	0.523	0.864
	Nonlinear	*q*_m_ (mg/g)	17.507	40.634	41.381	47.742
		*K*_L_ (L/mg)	0.303	0.667	0.395	0.785
		*R*^2^	0.989	0.965	0.557	0.873
		RMSE	0.436	2.029	1.621	2.032
		*R*^2^_adj_	0.987	0.959	0.468	0.847
Freundlich isotherm	Linear	1/*n*	0.413	0.511	0.553	0.829
		*K*_F_ (mg/g)(L/mg)^1/n^	4.253	15.129	29.813	40.004
		*R*^2^	0.997	0.982	0.985	0.983
	Nonlinear	1/*n*	0.354	0.479	0.524	0.957
		*K*_F_ (mg/g)(L/mg)^1/n^	4.412	15.480	31.110	38.418
		*R*^2^	0.995	0.984	0.982	0.978
		RMSE	0.283	1.388	1.556	2.302
		*R*^2^_adj_	0.994	0.981	0.979	0.973

For linear isotherm models, Langmuir maximum adsorption capacities (*q*_m_) of CP, CB, CZB, and ZCB were 16.207, 39.216, 41.041, and 47.349 mg/g, respectively, and Langmuir adsorption constants (*K*_L_) of CP, CB, CZB, and ZCB were 0.378, 0.770, 0.383 and 0.760 L/mg, respectively. For Freundlich isotherm, the 1/*n* values of CP, CB, CZB, and ZCB were 0.413, 0.511, 0.553, and 0.829, respectively. Freundlich adsorption constants (*K*_F_) of CP, CB, CZB, and ZCB were 4.253, 15.129, 29.813, and 40.004 (mg/g)(L/mg)^1/n^, respectively. For nonlinear isotherm models, Langmuir maximum adsorption capacities (*q*_m_) of CP, CB, CZB, and ZCB were 17.507, 40.634, 41.381, and 47.742 mg/g, respectively, and Langmuir adsorption constants (*K*_L_) of CP, CB, CZB, and ZCB were 0.303, 0.667, 0.395 and 0.785 L/mg, respectively. For Freundlich isotherm, the 1/*n* values of CP, CB, CZB, and ZCB were 0.354, 0.479, 0.524, and 0.957, respectively. Freundlich adsorption constants (*K*_F_) of CP, CB, CZB, and ZCB were 4.412, 15.480, 31.113, and 38.418 (mg/g)(L/mg)^1/n^, respectively.

For *R*^2^ value consideration, *R*^2^ values of CP, CB, CZB, and ZCB in linear Langmuir model were 0.971, 0.962, 0.523, and 0.864, respectively, and linear Freundlich model were 0.997, 0.982, 0.985, and 0.983, respectively. In addition, *R*^2^ values of CP, CB, CZB, and ZCB in nonlinear Langmuir model were 0.989, 0.965, 0.557, and 0.873, respectively, and nonlinear Freundlich model were 0.995, 0.984, 0.982, and 0.978, respectively. Moreover, *R*^2^_adj_ of CP, CB, CZB, and ZCB in nonlinear Langmuir model were 0.987, 0.959, 0.468, and 0.847, respectively, and *R*^2^_adj_ of CP, CB, CZB, and ZCB in nonlinear Freundlich model were 0.994, 0.981, 0.979, and 0.973, respectively. Since *R*^2^ values of CP, CB, CZB, and ZCB in both linear and nonlinear Freundlich models were higher than Langmuir models, their adsorption patterns corresponded to Freundlich isotherm associated with a physicochemical adsorption process. Therefore, a constant depicting of adsorption intensity (1/*n*) and Freundlich adsorption constant (*K*_F_) were used to consider lead adsorptions by chitosan materials. 0 < 1/*n* < 1 means favorable adsorption isotherm, and a high 1/*n* value refers the high lead adsorption capacity. *K*_F_ indicates the reach of the equilibrium lead adsorption^10^. Their 1/*n* and *K*_F_ values could be arranged in order from high to low of ZCB > CZB > CB > CP, so ZCB might remove lead higher than other chitosan materials corresponding to batch experiment results. Therefore, both adding ZnO and changing material form helped to improve chitosan material efficiencies, especially ZCB similarly reported by another study^21^.

Finally, since the equilibrium parameters and *R*^2^ values of CP, CB, CZB, and ZCB on lead adsorptions by linear and nonlinear Langmuir and Freundlich isotherm models had approximately close values, their results were consistent with each other. Therefore, both linear and nonlinear isotherm models were required to protect the data mistranslation^38–40^.

### Kinetic study

**Figure 7:**
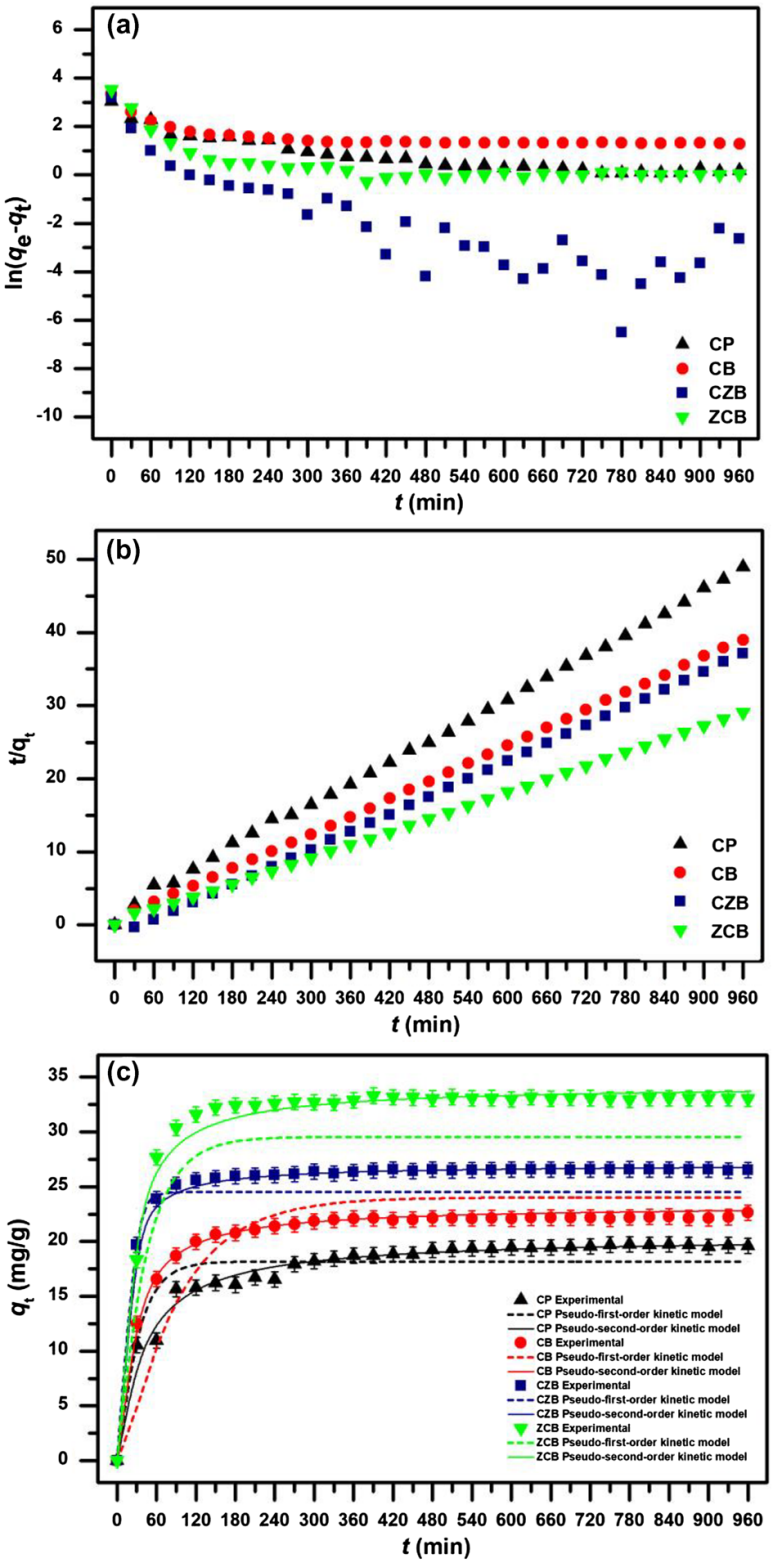
(**a**) Linear pseudo-first-order, (**b**) Linear pseudo-second-order, and (**c**) nonlinear kinetic models of chitosan powder (CP), chitosan beads (CB), chitosan beads mixed ZnO (CZB), and chitosan beads coated ZnO (ZCB) for lead adsorptions.

The adsorption rate and mechanism of all chitosan materials on lead adsorptions were explained by the kinetic study with the fitting of linear and nonlinear pseudo-first-order and pseudo-second-order kinetic models. Linear pseudo-first-order and pseudo-second-order kinetic models were plotted by ln(*q*_e_-*q*_t_) versus time (*t*) and *t/q*_t_ versus time (*t*) whereas nonlinear pseudo-first-order and pseudo-second-order kinetic models were plotted by *q*_t_ versus time (*t*). The results are demonstrated in Fig. 7a-c, and the adsorption kinetic parameters are reported in Table 4.

**Table 4:** The adsorption kinetic parameters of chitosan powder (CP), chitosan beads (CB), chitosan beads mixed ZnO (CZB), and chitosan beads coated ZnO (ZCB) for lead adsorptions.

Models	Regression method	Parameters	CP	CB	CZB	ZCB
Pseudo-first-order kinetic model	Linear	*q*_e_ (mg/g)	6.991	7.542	2.214	3.766
		*k*_1_ (min^-1^)	0.002	0.001	0.006	0.002
		*R*^2^	0.819	0.462	0.710	0.452
	Nonlinear	*q*_e_ (mg/g)	7.543	8.136	2.385	4.068
		*k*_1_ (min^-1^)	0.003	0.001	0.008	0.003
		*R*^2^	0.832	0.488	0.714	0.473
		RMSE	1.627	3.317	2.377	4.589
		*R*^2^_adj_	0.826	0.471	0.705	0.456
Pseudo-second-order kinetic model	Linear	*q*_e_ (mg/g)	20.450	24.752	24. 876	33.333
		*k*_2_ (g/mg·min)	0.001	0.003	0.004	0.007
		*R*^2^	0.999	1.000	1.000	1.000
	Nonlinear	*q*_e_ (mg/g)	20.491	25.020	25. 310	34.282
		*k*_2_ (g/mg·min)	0.001	0.002	0.002	0.004
		*R*^2^	0.981	0.997	0.996	0.979
		RMSE	0.547	0.271	0.286	0.924
		*R*^2^_adj_	0.980	0.996	0.996	0.978

For linear kinetic models, the adsorption capacities (*q*_e_) of a pseudo-first-order kinetic model of CP, CB, CZB, and ZCB were 6.991, 7.542, 2.214, and 3.766 mg/g, respectively, and the reaction rate constants of a pseudo-first-order kinetic model (*k*_1_) of CP, CB, CZB, and ZCB were 0.002, 0.001, 0.006, and 0.002 min^-1^, respectively. For a pseudo-second-order kinetic model, the adsorption capacities (*q*_e_) of CP, CB, CZB, and ZCB were 20.45, 24.752, 24.876, and 33.333 mg/g, respectively, and the reaction rate constants of a pseudo-second-order kinetic model (*k*_2_) of CP, CB, CZB, and ZCB were 0.001, 0.003, 0.004, and 0.007 g/mg·min, respectively. For nonlinear kinetic models, the adsorption capacities (*q*_e_) of a pseudo-first-order kinetic model of CP, CB, CZB, and ZCB were 7.543, 8.136, 2.385, and 4.068 mg/g, respectively, and the reaction rate constants of a pseudo-first-order kinetic model (*k*_1_) of CP, CB, CZB, and ZCB were 0.003, 0.001, 0.008, and 0.003 min^−1^, respectively. For a pseudo-second-order kinetic model, the adsorption capacities (*q*_e_) of CP, CB, CZB, and ZCB were 20.491, 25.020, 25.310, and 34.282 mg/g, respectively, and the reaction rate constants of a pseudo-second-order kinetic model (*k*_2_) of CP, CB, CZB, and ZCB were 0.001, 0.002, 0.002, and 0.004 g/mg·min, respectively.

For *R*^2^ value consideration, *R*^2^ values of CP, CB, CZB, and ZCB in linear pseudo-first-order kinetic model were 0.819, 0.462, 0.710, and 0.452, respectively, and linear pseudo-second-order kinetic model were 0.999, 1.000, 1.000, and 1.000, respectively. In addition, *R*^2^ values of CP, CB, CZB, and ZCB in nonlinear pseudo-first-order kinetic model were 0.832, 0.488, 0.714, and 0.473, respectively, and nonlinear pseudo-second-order kinetic model were 0.981, 0.997, 0.996, and 0.979, respectively. Moreover, *R*^2^_adj_ of CP, CB, CZB, and ZCB in nonlinear pseudo-first-order kinetic model were 0.826, 0.471, 0.705, and 0.456, respectively, and *R*^2^_adj_ of CP, CB, CZB, and ZCB in nonlinear pseudo-second-order kinetic model were 0.980, 0.996, 0.996, and 0.978, respectively. Since *R*^2^ values of CP, CB, CZB, and ZCB in both linear and nonlinear pseudo-second-order kinetic models were higher than pseudo-first-order kinetic models, their adsorption rate and mechanism of all chitosan materials corresponded to pseudo-second-order kinetic model relating to a chemisorption process with heterogeneous adsorption. Therefore, the adsorption capacity (*q*_e_) and pseudo-second-order kinetic rate constant (*k*_2_) were used to describe lead adsorptions of chitosan materials which high *q*_e_ and *k*_2_ values represent the high lead adsorption^14^. Their *q*_e_ and *k*_2_ values could be arranged in order from high to low of ZCB > CZB > CB > CP, so ZCB might remove lead higher than other chitosan materials corresponding to batch experiment results and adsorption isotherms. Therefore, it also confirmed that adding ZnO and changing material form improved chitosan material efficiencies, especially ZCB similarly reported by another study^21^.

Finally, the results of both linear and nonlinear pseudo-first-order and pseudo-second-order kinetic models of all chitosan materials were consistent with each other, it is also recommended to use both linear and nonlinear kinetic models to correctly data translations^38,41^.

### Probable mechanism of lead adsorptions on chitosan materials

**Figure 8:**
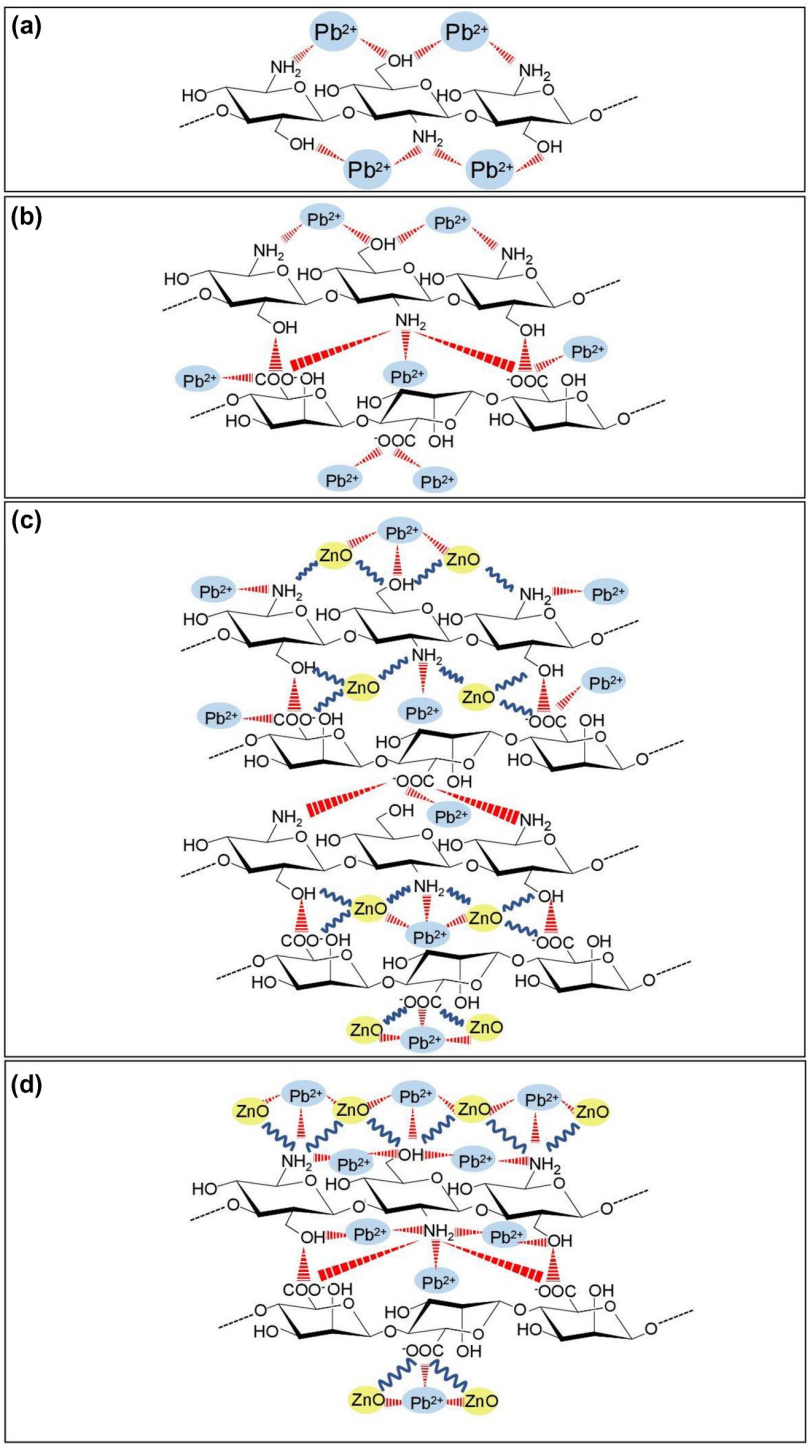
The possible mechanism for lead adsorptions by (**a**) chitosan powder (CP), (**b**) chitosan beads (CB), (**c**) chitosan beads mixed ZnO (CZB), and (**d**) chitosan beads coated ZnO (ZCB).

The possible mechanism of lead adsorptions on chitosan materials is demonstrated in Fig. 8a-d modified from E.H. Ablouh et al.^42^. The chemical functional groups on surface areas of chitosan materials might help to explain the probable mechanism of lead adsorptions in an aqueous solution, so their adsorption mechanisms might be upon which chemical compositions are in materials. For FTIR analysis, it detected the amine group (–NH_2_) and hydroxyl group (–OH) in all chitosan materials, and sodium alginate presented as carboxyl group (–COOH) was found in chitosan beads. In addition, ZnO was discovered in CZB and ZCB which interacted with –NH_2_ or –OH of chitosan materials. For CP, the main chemical functional groups of –NH_2_, and –OH were the main role in lead removal by the electrostatic interaction of replacing proton (H^+^) with lead (II) ions (Pb^2+^) shown in Fig. 8a. For CB, the adsorption of Pb^2+^ will occur by donating H^+^ of an amine group (–NH_2_) or hydroxyl group (–OH) in chitosan and carboxyl group (–COOH) of sodium alginate, and then Pb^2+^ are adsorbed by replacing H^+^ by the electrostatic interaction^30,43,44^ shown in Fig. 8b. For CZB, the main chemical functional groups of –NH_2_, –OH, –COOH, and ZnO were the main role in lead removal which ZnO might be connected to the chitosan chain and sodium alginate structure by forming of covalent and hydrogen bonds^45^. The adsorption of Pb (II) ions will occur similarly to CB shown in Fig. 8c. For ZCB, the main chemical functional groups of –NH_2_, –OH, –COOH, and ZnO were the main role in lead removal which ZnO might be coated on chitosan beads (CB) by covalent and hydrogen bonds, and the lead adsorption by ZCB was similarly to CB and CZB show in Fig. 8d.

## Conclusions

Four chitosan materials which were chitosan powder (CP), chitosan beads (CB), chitosan beads mixed ZnO (CZB), and chitosan beads coated ZnO (ZCB) were synthesized for lead adsorptions in an aqueous solution. CP was a fine yellow powdered color similar to the chitosan standard. CB was a yellow beaded color, and CZB and ZCB were a lightly yellow beaded color. ZCB demonstrated a higher size of surface area and a smaller pore size than other chitosan materials. CP represented the semi-crystalline structure, and CB represented the amorphous structure. For CZB and ZCB, they were semi-crystalline structures presenting the specific ZnO peaks. CP was a scaly-sheet and coarse surface whereas CB, CZB, and ZCB were sphere shapes with scaly-sheet surfaces. Moreover, ZnO was detected in CZB and ZCB. C, O, and N were the three main chemical elements in all chitosan materials, and Na, Ca, and Cl were found in CB, CZB, and ZCB. In addition, CZB and ZCB were also highly found Zn content. The main functional groups of chitosan materials were O–H, N–H, C–O, and –COOH representing sodium alginate in chitosan beads. In addition, ZnO interacted with NH_2_ or –OH in the chitosan and was detected in CZB and ZCB. The optimum conditions of CP, CB, CZB, and ZCB on lead adsorptions at 50 mg/L were 0.5 g, 14 h, pH 5, 0.4 g, 12 h, pH 5, 0.4, 12 h, pH 5, and 0.3, 12 h, pH 5, respectively. All chitosan materials could remove lead by more than 92%, and ZCB represented the highest lead removal efficiency of 98.91%. Therefore, both adding ZnO and changing material form improved chitosan material efficiencies. All chitosan materials corresponded Freundlich model and pseudo-second-order kinetic models associated with a physiochemical adsorption process. Therefore, all chitosan materials are potential materials for lead adsorptions in an aqueous solution whereas ZCB is the best option for industrial applications of wastewater treatment systems because of its high efficiency and no requirement of a separated section after treatment.

For future works, desorption experiments need to study the possible reuse of materials, and the column run experiments also are required for studying the possibly industrial applications in the case of a continuous flow system.

## Materials and methods

### Raw material

Shrimp shell wastes were obtained from Bang Lam Phu market, Khon Kaen province, Thailand, and then they were washed with tap water and distilled water to remove contaminants. Finally, they were dried in a hot air oven (Binder, FED 53, Germany) at 80 °C for 12 h and kept in a desiccator before use for the chitosan synthesis.

### Chemicals

All chemical reagents were analytical grades (AR) without purification. The commercial chitosan standard of ≥ 85% deacetylation was used as the chitosan standard (Sigma, Germany). For the chitosan synthesis, 2 N of 37% hydrochloric acid (HCl) (RCI Labscan, Thailand) and 2 N or 12.5 N of sodium hydroxide (NaOH) (RCI Labscan, Thailand) were used. For the bead formation, sodium alginate (C_6_H_9_NaO_7_) (Sigma, Germany) and calcium chloride (CaCl_2_) (KEMAUS, Australia) were used. In addition, zinc oxide (ZnO) (QR $\ddot{e}$ C, New Zealand) was used for adding ZnO into chitosan materials. Lead nitrate (Pb(NO_3_)_2_) (QR $\ddot{e}$ C, New Zealand) was used for preparing initial lead concentrations of the water sample. For pH adjustments, 0.65% nitric acid (HNO_3_) (Merck, Germany), and 1% sodium hydroxide (NaOH) (RCI Labscan, Thailand) were used.

### Material synthesis

#### The synthesis of chitosan powder (CP)

**Figure 9:**
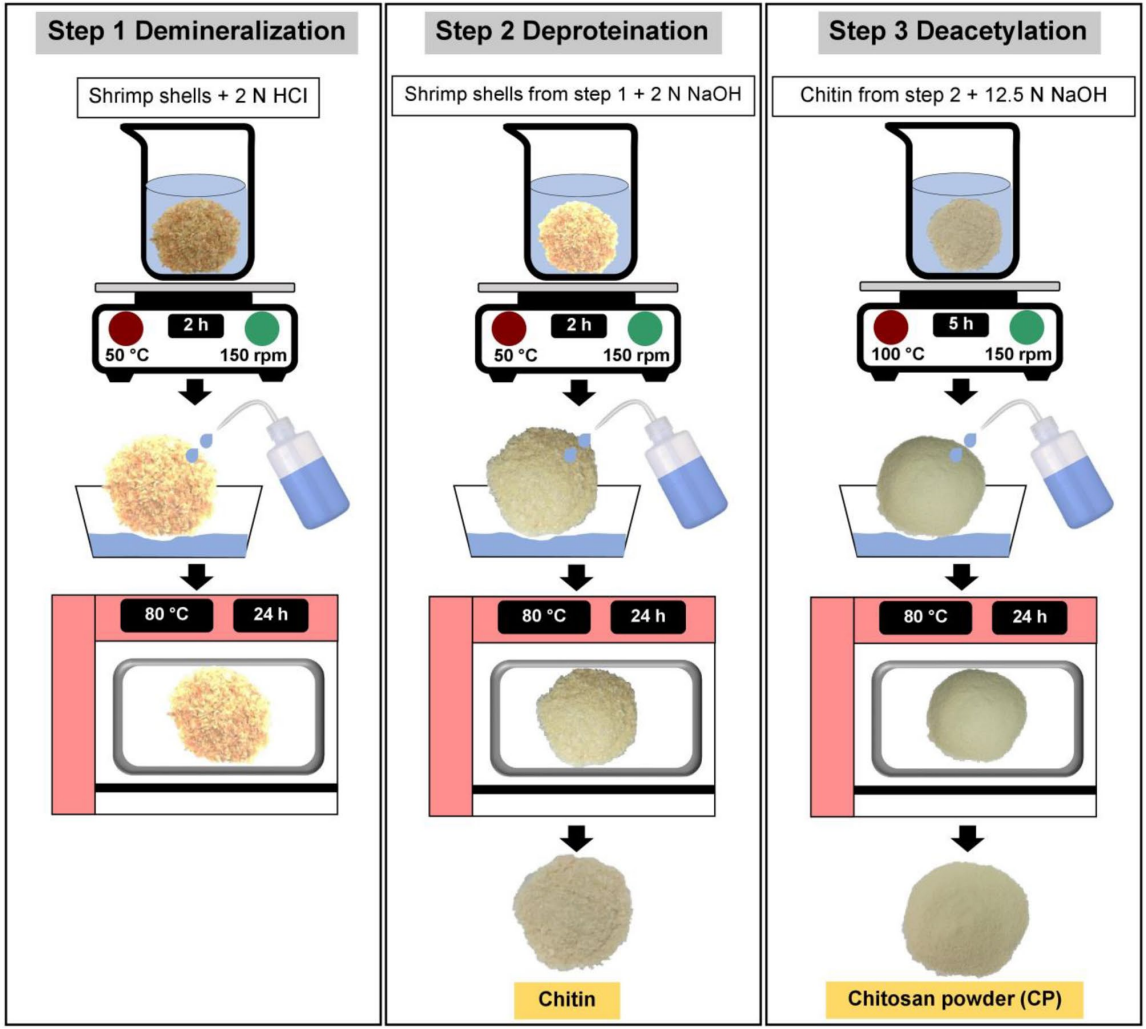
Three processes of chitosan synthesis.

The chitosan synthesis included three processes which were demineralization, deproteination, and deacetylation shown in Fig. 9, and the details were clearly explained below:

*Step 1 (Demineralization)* The ratio of 1:20 (w/v) of shrimp shells and 2 N HCI was applied, so 10 g of shrimp shells and 200 mL of 2 N HCI were added to a 1000 mL Beaker. Then, the sample was mixed on a hot plate (Ingenieurb $\ddot{\mathrm{u}}$ ro CAT, M. Zipperer GmbH, M 6, Germany) at 50 °C with a constant stirrer of 150 rpm for 2 h. After that, the sample was washed with distilled water until obtaining the natural pH and dried in a hot air oven at 80 °C for 24 h.

*Step 2 (Deproteination)* The ratio of 1:20 (w/v) of shrimp shells from step 1 and 2 N NaOH was applied, so 10 g of shrimp shells and 200 mL of 2 N NaOH were added to a 1000 mL Beaker. Then, the sample was mixed on a hot plate at 50 °C with a constant stirrer of 150 rpm for 2 h. After that, the sample was washed with distilled water until obtaining the natural pH and dried in a hot air oven at 80 °C for 24 h. The sample was called chitin and kept in a desiccator before use.

*Step 3 (Deacetylation)* The ratio of 1:20 (w/v) of chitin from step 2 and 12.5 N NaOH was applied, so 10 g of chitin and 200 mL of 12.5 N NaOH were added to a 1000 mL Beaker. Then, the sample was mixed on a hot plate at 100 °C with a constant stirrer of 150 rpm for 5 h. After that, the sample was washed with distilled water until obtaining the natural pH and dried in a hot air oven at 80 °C for 24 h. The sample was called chitosan, and then the sample was ground in a blender and sieved at 125 µm. Finally, the sample was kept in a desiccator before use and called chitosan powder (CP).

**Figure 10:**
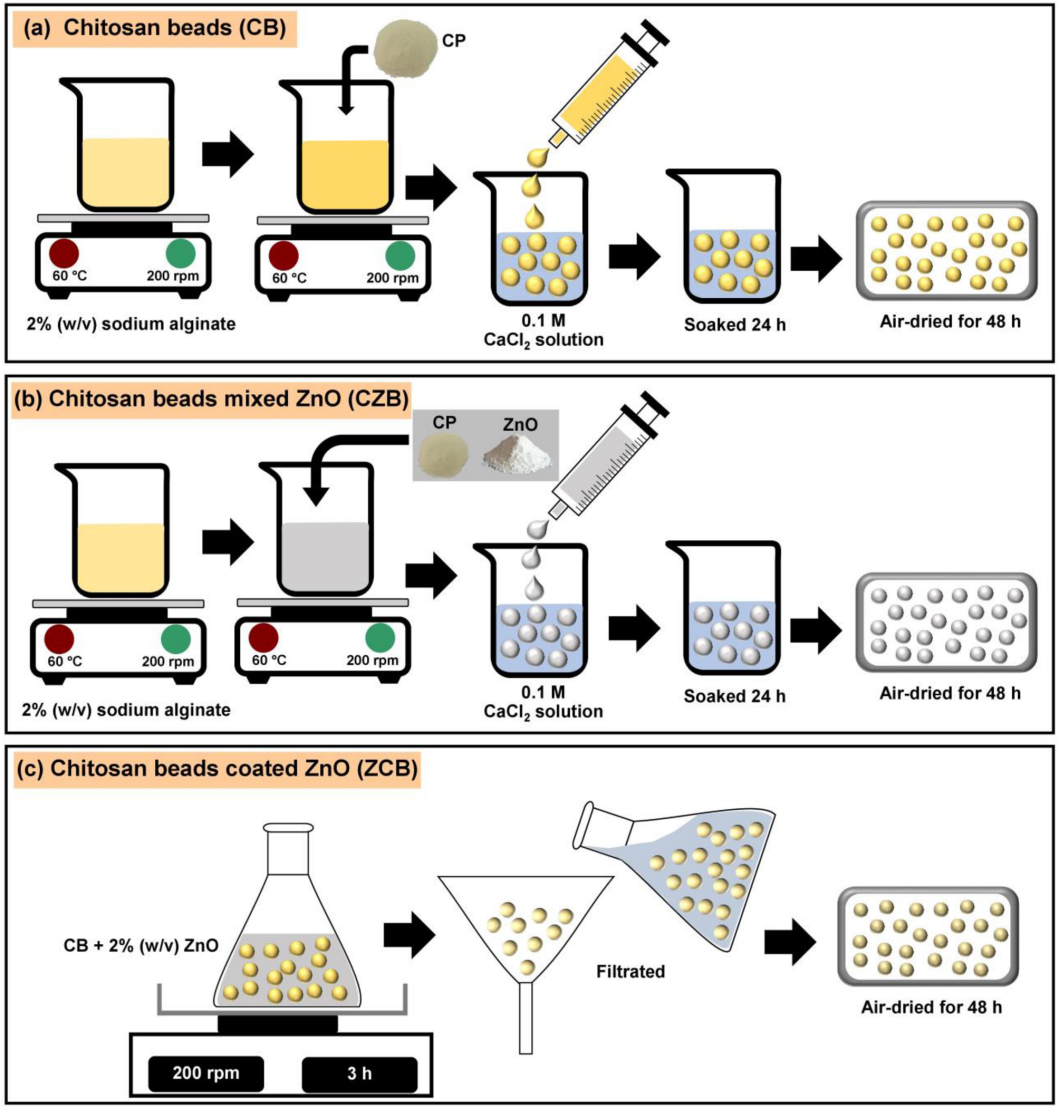
The synthesis methods of (**a**) chitosan beads (CB), (**b**) chitosan beads mixed ZnO (CZB), and (**c**) chitosan beads coated ZnO (ZCB).

The synthesis methods of chitosan beads (CB), chitosan beads mixed ZnO (CZB), and chitosan beads coated ZnO (ZCB) are demonstrated in Fig. 10a–c, and the details were clearly explained below:

#### The synthesis of chitosan beads (CB)

4 g of CP were added to 200 mL of 2% sodium alginate, and then they were homogeneously mixed and heated by a hot plate at 60 °C with a constant stirring of 200 rpm. Then, the sample was dropped by drop by using a 10 mL syringe into 250 mL of 0.1 M CaCl_2_. The beaded samples were soaked in 0.1 M CaCl_2_ for 24 h, and then they were filtered and rinsed with deionized water. Finally, they were air-dried for 48 h and kept in a desiccator until use. The beaded samples were called chitosan beads (CB).

#### The synthesis of chitosan beads mixed ZnO (CZB)

4 g of CP and 4 g of ZnO were added to 200 mL of 2% sodium alginate, and then they were homogeneously mixed and heated by a hot plate at 60 °C with a constant stirring of 200 rpm. Then, the sample was dropped by drop by using a 10 mL syringe into 250 mL of 0.1 M CaCl_2_. The beaded samples were soaked in 0.1 M CaCl_2_ for 24 h, and then they were filtered and rinsed with deionized water. Finally, they were air-dried for 48 h and kept in a desiccator until use. The beaded samples were called chitosan beads mixed ZnO (CZB).

#### The synthesis of chitosan beads coated ZnO (ZCB)

4 g of CP were added to 200 mL of 2% sodium alginate, and then they were homogeneously mixed and heated by a hot plate at 60 °C with a constant stirring of 200 rpm. Then, the sample was dropped by drop by using a 10 mL syringe into 250 mL of 0.1 M CaCl_2_. The beaded samples were soaked in 0.1 M CaCl_2_ for 24 h, and then they were filtered, rinsed with deionized water, and air-dried for 48 h. The dried beaded samples were added to 200 mL of 2% ZnO, and then the samples were shaken by an orbital shaker (GFL, 3020, Germany) of 200 rpm for 3 h. After that, the samples were filtrated and air-dried for 48 h. Finally, they were kept in a desiccator until use. The beaded samples were called chitosan beads coated ZnO (ZCB).

**Figure 11:**
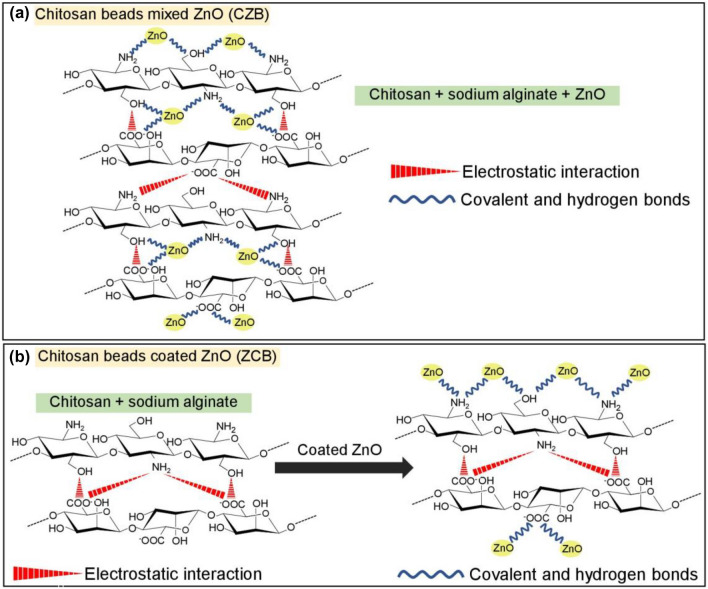
The formation schemes of (**a**) chitosan beads mixed ZnO (CZB) and (**b**) chitosan beads coated ZnO (ZCB).

The formation schemes of CZB and ZCB are demonstrated in Fig. 11a,b. For CZB, the chitosan powder (CP), ZnO, and sodium alginate were homogenously mixed and formed the complex compound to be CZB. ZnO might be connected to amine (–NH_2_) or hydroxyl group (–OH) of chitosan and carboxyl group (–COOH) of sodium alginate by covalent and hydrogen bonds. In addition, the carboxyl group (–COOH) of sodium alginate was connected to amine (–NH_2_) or hydroxyl group (–OH) of chitosan by the electrostatic interaction^19,32,46,47^ shown in Fig. 11a. For ZCB, CP and sodium alginate were homogeneously mixed for forming chitosan beads (CB) which the carboxyl group (–COOH) of sodium alginate was connected to amine (–NH_2_) or hydroxyl group (–OH) of chitosan by the electrostatic interaction^19,32^. Then, ZnO was coated on CB and formed the complex compound of chitosan, sodium alginate, and ZnO to be ZCB which ZnO might be connected to the amine (–NH_2_) or hydroxyl group (–OH) of chitosan and the carboxyl group (–COOH) of sodium alginate by covalent and hydrogen bonds^46,47^ shown in Fig. 11b.

### Characterization of chitosan materials

All chitosan materials were characterized to investigate the size of surface area, pore volume, pore size, crystalline formation, surface morphology, chemical compositions, and chemical functional groups. The size of surface area, pore volume, and pore size of chitosan materials were analyzed by Brunauer–Emmett–Teller (BET) (Quantachrome instruments, QuadraSorb evo, USA) with nitrogen gas (N_2_) adsorption–desorption isotherm at 77. 3 K and degas temperature of 80 ℃ for 6 h. The crystalline formation of chitosan materials was identified by X-ray Diffractometer (XRD) (Bruker, D8 Advance, Switzerland) in a range of 2 $\uptheta$ = 5–80 $^\circ$. Field Emission Scanning Electron Microscopy and Focus Ion Beam (FESEM-FIB) with Energy Dispersive X-ray Spectrometer (EDX) (FEI, Helios NanoLab G3 CX, USA) was used for studying the surface morphology and chemical compositions of chitosan materials which the samples were mounted on aluminum stubs by double-side carbon tapes and coated with by gold-coater for 4 min by using 108 auto Sputter Coater with thickness controller MTM-20 model (Cressington, Ted Pella Inc, USA) with analyzing at 10 kV accelerating voltage. Finally, the chemical functional groups of chitosan materials were classified by Fourier Transform Infrared Spectroscopy (FTIR) (Bruker, TENSOR27, Hong Kong) in a range of 4000–600 cm^−1^ with a resolution of 4 cm^−1^ and 16 scans over the entire covered range.

### Batch experiments

A series of batch experiments were designed for investigating lead removal efficiencies of chitosan materials with various doses, contact time, pH, and concentration, and lead concentrations were analyzed by Atomic Absorption Spectrophotometer (AAS) (PerkinElmer, PinAAcle 900 $^\circ$ F, USA). The details of batch experiments were clearly explained below:

#### Effect of dose

The different doses of chitosan materials from 0.1 to 0.5 g were used to investigate lead removal efficiencies with the control condition of the initial lead concentration of 50 mg/L, a sample volume of 200 mL, a shaking speed of 150 rpm, a contact time of 24 h, and pH 5.

#### Effect of contact time

The optimum dose and the contact time from 2 to 24 h were used to study lead removal efficiencies of chitosan materials with contact time effect. The control condition was the initial lead concentration of 50 mg/L, a sample volume of 200 mL, a shaking speed of 150 rpm, and pH 5.

#### Effect of pH

The optimum dose and contact time were used to investigate lead removal efficiencies of chitosan materials with pH effect. The pH values of 1, 3, 5, 7, 9, and 11 were used as representative acid, neutral, and base conditions for studying pH effects on chitosan materials. The control condition was the initial lead concentration of 50 mg/L, a sample volume of 200 mL, and a shaking speed of 150 rpm.

#### Effect of concentration

The optimum dose, contact time, and pH of chitosan materials, and initial lead concentrations from 10 to 70 mg/L were examined for lead removal efficiencies with concentration effect. The control condition was a sample volume of 200 mL and a shaking speed of 150 rpm.

Triplicate experiments were conducted and reported the average value, and lead removal efficiency in the percentage is calculated following Eq. (1):

1 $${\text{Lead}}\;{\text{ removal }}\;{\text{efficiency\,}}\left( \% \right) \, = \, ((C_{0} - C_{e} )/C_{0} ) \times \, 100$$

where *C*_e_ is the equilibrium of lead concentration (mg/L), and *C*_0_ is the initial lead concentration *(*mg/L).

### Adsorption isotherms

The adsorption pattern of chitosan materials was explained by using linear and nonlinear Langmuir and Freundlich isotherms following Eqs. (2–5):

Langmuir isotherm^48^:

2 $${\text{Linear:}}\, C_{e} /q_{e} = 1/q_{m} K_{L} + C_{e} /q_{m}$$

3 $${\text{Nonlinear:}}\,q_{e} = \, q_{m} K_{L} C_{e} /1 + K_{L} C_{e}$$

Freundlich isotherm^49^:

4 $${\text{Linear:}}\, \log q_{e} = \log K_{F} + 1/n\log C_{e}$$

5 $${\text{Nonlinear:}}\,q_{e} = \, K_{F} C_{e}^{1/n}$$

where *q*_e_ is the capacity of lead adsorption on chitosan materials at equilibrium (mg/g), *q*_m_ is the maximum amount of lead adsorption on chitosan materials (mg/g), *C*_e_ is the equilibrium of lead concentration (mg/L*)*, *K*_L_ is Langmuir adsorption constant (L/mg), *K*_F_ is Freundlich constant of adsorption capacity (mg/g)(L/mg)^1/n^, and *n* is the constant depicting of the adsorption intensity. Graphs of linear Langmuir and Freundlich isotherms were plotted by *C*_e_*/q*_e_ versus *C*_e_ and log *q*_e_ versus log *C*_e_, respectively whereas graphs of nonlinear Langmuir and Freundlich isotherms were plotted by *q*_e_ versus *C*_e_*.*

For the adsorption isotherm experiment, the optimum dose of chitosan materials was applied with various initial lead concentrations from 10 to 70 mg/L with the control condition of a water sample of 200 mL, a contact time of 12 h, pH 5, and a shaking speed of 150 rpm.

### Adsorption kinetics

The adsorption kinetic was investigated for explaining the adsorption rate and mechanism of chitosan materials by using linear and nonlinear pseudo-first-order and pseudo-second-order kinetic models following Eqs. (6–9):

Pseudo-first-order kinetic model^50^:

6 $${\text{Linear:\,}}\ln (q_{e} - q_{t} ) = \ln q_{e} {-}k_{1} t$$

7 $${\text{Nonlinear:}}\,q\mathrm{t} = q\mathrm{e}(1-{e}^{{-k}_{1}t})$$

Pseudo-second-order kinetic model^51^:

8 $${\text{Linear:\,}}t/q_{t} = 1/k_{2} q_{e}^{2} + \, (1/q_{e} )t$$

9 $${\text{Nonlinear:\,}}q_{t} = \, k_{2} q_{e}^{2} t/(1 + \, q_{e} k_{2} t)$$

where *q*_e_ (mg/g) and *q*_t_ (mg/g) are the capacities of lead adsorbed by chitosan materials at equilibrium and at the time (*t*), respectively. *k*_1_ (min^-1^) and *k*_2_ (g/mg∙min) are the reaction rate constants of pseudo-first-order and pseudo-second-order kinetic models, respectively. Graphs of linear pseudo-first-order and pseudo-second-order kinetic models were plotted by ln(*q*_e_-*q*_t_) versus time (*t*) and *t/q*_t_ versus time (*t*), respectively whereas graphs of nonlinear pseudo-first-order and pseudo-second-order kinetic models were plotted by the capacity of lead adsorbed by chitosan materials at the time (*q*_t_) versus time (*t*).

For the adsorption kinetic experiment, the optimum dose of chitosan materials was applied with the control condition of the initial lead concentration of 50 mg/L, a sample volume of 1000 mL, a contact time of 16 h, pH 5, and a shaking speed of 150 rpm.

## Data Availability

The datasets used and/or analyzed during the current study are available from the corresponding author upon reasonable request.
